# Effect of G-Quadruplex Polymorphism on the Recognition of Telomeric DNA by a Metal Complex

**DOI:** 10.1371/journal.pone.0058529

**Published:** 2013-03-13

**Authors:** Caterina Musetti, A. Paul Krapcho, Manlio Palumbo, Claudia Sissi

**Affiliations:** 1 Department of Pharmaceutical and Pharmacological Sciences, University of Padova, Padova, Italy; 2 Department of Chemistry, University of Vermont, Burlington, Vermont, United States of America; Florida International University, United States of America

## Abstract

The physiological role(s) played by G-quadruplexes renders these ‘non-canonical’ DNA secondary structures interesting new targets for therapeutic intervention. In particular, the search for ligands for selective recognition and stabilization of G-quadruplex arrangements has led to a number of novel targeted agents. An interesting approach is represented by the use of metal-complexes, their binding to DNA being modulated by ligand and metal ion nature, and by complex stoichiometry. In this work we characterized thermodynamically and stereochemically the interactions of a Ni(II) bis-phenanthroline derivative with telomeric G-quadruplex sequences using calorimetric, chiroptical and NMR techniques. We employed three strictly related sequences based on the human telomeric repeat, namely Tel22, Tel26 and wtTel26, which assume distinct conformations in potassium containing solutions. We were able to monitor specific enthalpy/entropy changes according to the structural features of the target telomeric sequence and to dissect the binding process into distinct events. Interestingly, temperature effects turned out to be prominent both in terms of binding stoichiometry and ΔH/ΔS contributions, while the final G-quadruplex-metal complex architecture tended to merge for the examined sequences. These results underline the critical choice of experimental conditions and DNA sequence for practical use of thermodynamic data in the rational development of effective G-quadruplex binders.

## Introduction

The potential of nucleic acids to fold into non canonical secondary structures and the assessment of their role in regulating physiological processes is increasingly becoming object of interest [1]–[3]. Conformation is generally strictly related to DNA sequence but DNA-protein interactions can largely affect it [4], [5]. Additionally, changes in ionic strength, salt composition, pH as well as interactions with small molecules can drive nucleic acid structural transitions and trap a given sequence in a specific energetically favored conformation.

A relevant example of highly ordered DNA structures is provided by G-quadruplexes, peculiar structural arrangements which can be assumed by G-rich sequences [6]. They derive from the overlapping of planar arrays in which four guanines are paired together through a network of Hoogsteen bonds and are further stabilized by the presence of a monocationic atom (mainly Na^+^ or K^+^) [7].

Genome-wide analysis reveals that G-rich sequences are not randomly distributed along the genome but are clustered in specific regions which mainly correspond to gene promoters [8], [9]. This finding suggested the structural equilibrium duplex-quadruplex as an additional level of control of protein expression. Since often they encode for oncogenes, selective induction of G-quadruplex structures by small ligands has been investigated as a novel chemotherapeutic approach [10]. An additional G-enriched site is represented by telomeres which form the termini of the chromosomes and, in humans, are composed of repetitive TTAGGG sequences oriented 5′ to 3′ [11]. They are involved in maintaining chromosomal stability and genome integrity [12], [13]. Interestingly, in over 85% of human cancer cells the cell ability to indefinitely replicate and become immortal is achieved through the activation of telomerase, a RNA-dependent DNA polymerase responsible for telomere elongation. Telomerase is inactive in somatic cells, thus it has become an attractive target for anticancer therapy. Among different possible approaches, we are particularly interested in indirect inhibition of telomerase activity [14], [15]. Indeed, the peculiar G-quadruplex conformational arrangement that the G-rich telomeric sequence can assume is not recognized by the enzyme which processes only a single stranded template. For this reason, small molecules able to induce and stabilize the G-quadruplex form of the telomeric sequence can prevent the hybridization of the telomerase RNA template onto the primer and thus inhibit the enzymatic activity [16]. Accordingly, G-quadruplex induction has been confirmed to stimulate cellular senescence, apoptosis or autophagy [17]–[19]. Additionally, modification of the telomere structural equilibria can further alter the recognition by telomere-directed proteins leading to short term cytotoxic effects [15].

Up-to date, most of the reported G-quadruplex binders are ligands structurally based on extended planar aromatic arrays in which the π-delocalized system allows stacking interactions with the external guanine tetrads. Generally, ligand scaffolds are further functionalised with positively charged side chains that enhance the ligand affinity by interacting with the negative phosphates of the DNA backbone [20], [21].

Although the majority of these compounds are organic systems, recently metal ion complexes were elegantly used as potential G-quadruplex binders and stabilizers [22]–[25]. In these complexes, the metal center binds to specific ligands according to the electronic configuration of the species involved, which ultimately produces specific geometries around the coordination sphere [26], [27]. Additionally, the coordination of the metal to aromatic ligands (such as canonical G-quadruplex ligands) can withdraw the electron density yielding an electron-deficient system with increased π–π interacting capabilities towards the G-quartet planes. As a result, the G-quadruplex stabilization properties of the metal complex are distinct from those of the ligand itself due to the charged metal center and the stoichiometry/stereochemistry of the metal complex arrangement.

Phenanthroline is a known efficient ligand for several metal ions able to form metal complexes of different geometries. In particular, extensive literature data describes the ability of the phenanthroline moiety to interact with duplex DNA with characteristic affinity and binding mode when bound to metal ions [26].

In previous works, we exported this model toward G-quadruplex DNA with the idea that only selected metal ions could coordinate phenanthroline units according to geometries suitable to enhance the G-quadruplex recognition and disfavor the binding to the double helix [28], [29]. In agreement with other authors [30], our investigation showed Cu(II) and Ni(II) as the most effective transition metals in providing phenanthroline complexes selective toward G-quadruplex [28], [29]. Moreover, the evaluation of several phenanthroline-based free ligands and of their Ni(II) complexes indicated the assembly of two phenanthroline moieties around the metal center as the key structural feature required to enhance such a selectivity. Based on these results, we proposed a model according to which the extended planar surface achieved through metal coordination promotes stacking onto a guanine quartet. In particular, we suggested the terminal G-tetrads as the potential DNA ligand binding sites.

**Figure 1:**
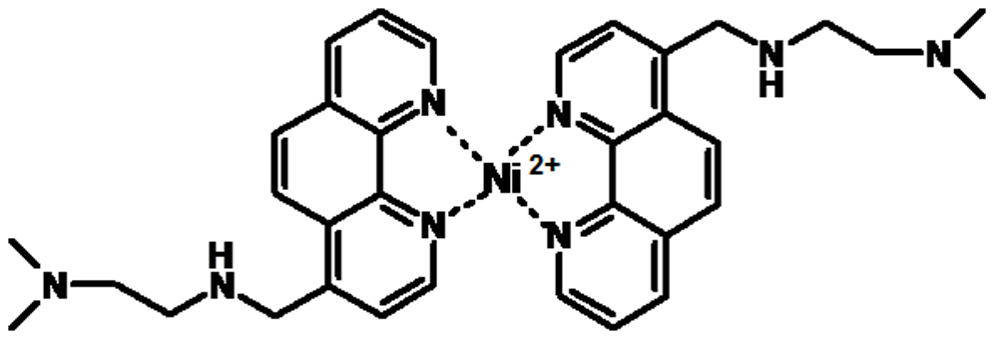
Tested metal complex (K34)_2_Ni(II). Structural representation of the metal complex (K34)_2_Ni(II).

To investigate in more details this binding mode, we decided to perform a thermodynamic characterization of the interactions of these metal complexes with the telomeric G-quadruplex. In particular, here we focus on the Ni(II) complex formed by the phenathroline derivative K34, (K34)_2_Ni(II) (Figure 1). Indeed this metal complex has physical and chemical properties, such as water solubility and thermal stability, which make it a good candidate for biologically relevant studies.

Our previous target sequence was the telomeric sequence Tel22. However, in potassium containing solution, its G-quadruplex form is highly polymorphic [31]. This property can affect the quantitative description of the ligand binding process. Distinctly, NMR studies reveal that two related sequences wtTel26, and Tel26 in the same experimental conditions assume prevalent well-defined different hybrid forms [32], [33]. Noteworthy, these sequences comprise the reference sequence Tel22 but they contain additional 5′ and 3′-flanking dinucleotides which are involved in pairing and stacking interactions thus providing capping structures which lock DNA preferentially in one conformation [34]. For this reason, we performed calorimetric and circular dichroism titrations using all these three sequences based on the telomeric one: the reference Tel22, wtTel26 and Tel26.

Using this approach we were able not only to monitor different ligand-DNA affinities according to the structural features of the target telomeric sequence but also to dissect the binding process into distinct events.

## Materials and Methods

### Oligonucleotides solutions

Lyophilized synthetic oligonucleotides were purchased from Metabion International AG (Martinsried, Germany). The sequences were: Tel22 (5′-AGGGTTAGGGTTAGGGTTAGGG-3′), wtTel26 (5′-TTAGGGTTAGGGTTAGGGTTAGGGTT-3′), Tel26 (5′-AAAGGGTTAGGGTTAGGGTTAGGGAA-3′) and scT22 (5′-GGATGTGAGTGTGAGTGTGAGG -3′).

Before each experiment, G-quadruplex forming sequences were dissolved in 10 mM Tris, 20 mM KCl at pH 7.5, extensively washed with the same buffer using Millipore Filter Units (MWCO: 3000), diluted to the desired concentration, heated at 95°C for 5 minutes and slowly cooled to room temperature to insure folding of the sequence. Oligonucleotides concentration was determined by UV-Vis from absorbance at 260 nm using their reported molar extinction coefficient.

### Ligand, metal ion and metal complex solutions

K34 was synthesized as previously reported [29]. Stock solutions (4 mM) were prepared in 10 mM Tris, 20 mM KCl at pH 7.5 and diluted to the required concentrations with the same buffer. NiCl_2_ (0.8 M) was dissolved in deionized water and metal ion concentration was determined by ICP (Inductively Coupled Plasma, Optima 3000 DV Perkin Elmer). This solution was further diluted in 10 mM Tris, 20 mM KCl at pH 7.5. Solutions of (K34)_2_Ni(II) were prepared by mixing the required volumes of ligand and metal ion solutions.

### Circular Dichroism Studies

Circular dichroism spectra were recorded on a Jasco J-810 spectropolarimeter equipped with a Peltier temperature controller in 10 mM Tris, 20 mM KCl at pH 7.5 using a 10 mm path-length cell. Before data acquisition, DNA solutions (4 µM strand concentration) were heated at 95°C for 5 min and left to cool down at room temperature over night (o.n.). Each reported spectrum represents the average of 2 scans recorded with 1-nm step resolution. Observed CD signals were converted to mean residue ellipticity [Θ]  =  deg x cm^2^× dmol^−1^ (Mol. Ellip.).

For scT22, the saturation fraction f (ΔCD/ΔCD_max_) was analyzed according to equation (1) 
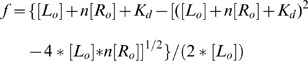
(1)


where L_o_ and R_o_ are the ligand and DNA concentration expressed in residues, respectively, and *n* is the complex stoichiometry.

Thermal denaturation experiments were performed by recording the optical signal while increasing the temperature at 0.8°C/min. The melted solution was then cooled down at the same temperature change rate to check for hysteresis. Melting temperatures (Tm) were calculated from the first derivatives of the melting profiles. Each curve was repeated at least three times and errors were±0.4°C. ΔTm were calculated by subtracting the Tm value recorded in the presence of the ligand from the corresponding value in the absence of ligand.

### Isothermal Titration Calorimetry (ITC)

ITC titrations were performed on a MicroCal VP ITC instrument in 10 mM Tris, 20 mM KCl at pH 7.5 at 25°C and 37°C. Working solutions were degassed for 5 minutes prior the use. Volumes of 10 µl of K34 (1 mM) or (K34)_2_Ni(II) (0.5 mM) were injected into a solution of previously folded DNA (25 µM). The ITC titration settings were: injection volume 10 µl, spacing between ligand injection 360 s, injection time 10 s, stirring speed 345 rpm, equilibration time 60 s.

Before data analysis, raw data were corrected for the heat of dilution. Heats were integrated and binding parameters were calculated according to one or two binding site model using Origin Software.

Data analysis provides ΔH (reaction enthalpy change, kcal. mol^−1^), K_a_ (binding constant, M^−1^), and *n* (number of bound ligands) whereas the Gibbs energy and the entropic contribution were calculated using the relationships ΔG  =  −RT ln K_a_ and ΔG  =  ΔH − TΔS, respectively.

For the interaction of our metal complex with Tel26 at 25°C the best fitting results were obtained by a two sequential binding sites model always. According to it the number of sequential sites must be exactly integral and thus it is held constant during the fitting procedure.

### Nuclear Magnetic Resonance (NMR)

NMR experiments were performed at 25 °C on a Bruker DMX 600 spectrometer, equipped with a 5 mm TXI probe with gradients, and the data were processed using the TOPSPIN 2.0 software.

Samples were prepared in 90%/10% H_2_O/D_2_O solution. NMR samples contained 0.17 mM DNA in 20 mM K-phosphate buffer, pH 7.5 in the presence/absence of (K34)_2_Ni(II) at 2∶1 ligand:DNA molar ratio.

1H one-dimensional spectra were acquired with 32 k scans, 1.52 s acquisition time, 1 s relaxation delay, 18 ppm spectral width.

Suppression of the water signal was achieved using the WATERGATE sequence before acquisition. For proton assignments see [32], [33].

## Results

### ITC Based Thermodynamic Studies

To obtain information on the binding of (K34)_2_Ni(II) to the G-quadruplex telomeric sequence we performed Isothermal Titration Calorimetry (ITC) analysis which provides a direct evaluation of thermodynamic parameters relevant to describe biomolecular interactions [35], [36]. As previously reported, this metal complex was shown to be stable in our working conditions [29] and did not tend to dissociate as evidenced by spectroscopic titrations (Figure S1). To evaluate the contribution of DNA structural arrangements in the binding process the analysis was performed with Tel22 and, in addition, with Tel26 and wtTel26 which preferentially assume a Hybrid 1 and Hybrid 2 folding, respectively [32]–[34]. These two G-quadruplex forms share common structural elements such as the overlapping of three G-tetrads and a mixed parallel/antiparallel orientation of the four strands. However, they differ for the loops arrangement and the relative strand orientation. Moreover, specific capping structures are formed. In particular, a T∶A∶T triple capping is present in the Hybrid 2 structure while an A∶T base pair and an adenine triple capping are found in the Hybrid 1 arrangement [32], [33].

**Figure 2:**
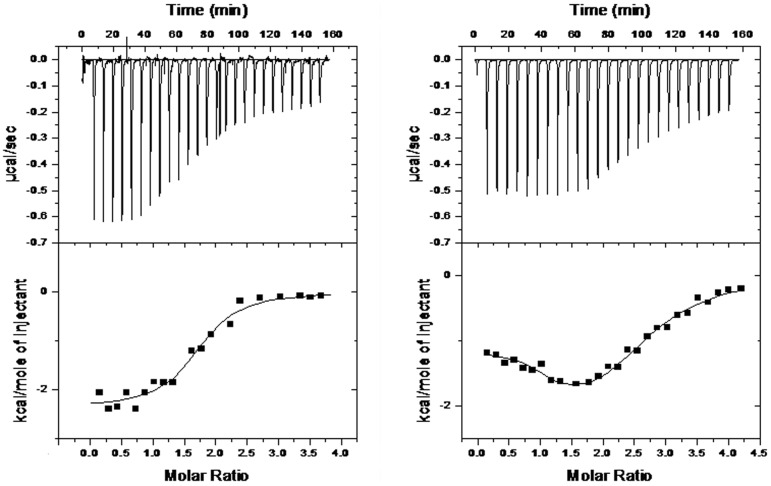
(K34)_2_Ni(II) shows different binding profiles towards wtTel26 and Tel26 at 25°C. ITC profiles corresponding to the titration of 25 µM wtTel26 (Panel A) or Tel26 (Panel B) with (K34)_2_Ni(II) at 25°C in 10 mM Tris, 20 mM KCl, pH 7.5. Raw ITC data (top panel) and binding isotherms (bottom panel).

In our experimental conditions, addition of (K34)_2_Ni(II) to any tested G-quadruplex folded sequences resulted in heat release which indicates the occurrence of an exothermic process (Figure 2).

At 25°C, data collected in the presence of Tel22 were poorly reproducible, thus, unfortunately, they could not be used for a safe comparison. Nevertheless data acquired with Tel26 and wtTel26 underlined interesting features of the (K34)_2_Ni(II) binding process which share several analogies but also quite peculiar differences as a function of the sequence of the nucleic acid target.

Recognition of the wtTel26 sequence (largely folded in a Hybrid 2 arrangement), showed a sigmoidal profile consistent with a single set of binding sites (Figure 2A). Data were analyzed accordingly, thus assuming any potential site of interaction as equivalent and independent. Such an analysis fits well the experimental data and indicates the binding of two metal complexes per G-quadruplex molecule. The resulting best-fit thermodynamic parameters are reported in Table 1.

**Table 1:** Thermodynamic parameters derived from ITC titrations describing the interaction of (K34)_2_Ni(II) with Tel26 and wtTel26 at 25°C in 10 mM Tris, 20 mM KCl, pH 7.5.

	wtTel26	Tel26	
n (ligands per G4)	1.8±0.1	1*	1*
Ka*10^−5^ (M^−1^)	4.7±1.7	8.7±3.3	0.3±0.1
ΔH (kcal*mol^−1^)	−2.4±0.1	−1.8±0.14	−7.5±0.3
ΔS (cal*mol^−1^ *K^−1^)	17.9	21.1	−5.0
-TΔS (kcal*mol^−1^)	−5.3	−6.3	1.5
ΔG (kcal*mol^−1^)	−7.7±0.2	−8.1±0.2	−6.0±0.1

* : the applied model of sequential binding sites considers the binding events as exactly integral numbers. Reported data are the average of three independent measurements.

The calorimetric titration of Tel26 (folded in a Hybrid 1 arrangement) with the same ligand (K34)_2_Ni(II) provided a binding isotherm, derived from the integrated heat data, which corresponds to two distinct binding events (Figure 2B). Data analysis indicated that the best fitting was obtained using a two sequential binding model. In particular the experimentally recorded profile is well described by two sequential processes, each resultant from the interaction of one metal complex to one DNA target. Thus, a final stoichiometry of two (K34)_2_Ni(II) molecules associated to one G-quadruplex template was confirmed. Thermodynamic parameters (Table 1) describing the first binding event showed a Ka of 8.66*10^5^ M^−1^ which is higher, albeit of the same order of magnitude, in comparison to the one observed with wtTel26. The binding of the second metal complex is characterized by a lower binding constant associated to a larger favourable enthalpy change accompanied by a negative ΔS value.

By increasing the working temperature up to 37°C, the binding model of (K34)_2_Ni(II) towards wtTel26 is essentially conserved (Figure S2A). Although two temperature data points are not sufficient to properly analyze the d(ΔH)/dT relationship, the negative temperature dependence of ΔH points to a negative ΔCp, which can be related to a hydrophobic effect or to the occurrence of temperature dependent conformational equilibria [37], [38]. Interestingly, the binding affinity and, consequently, the resulting negative free energy are not largely affected by changes in the working temperature (Table 2). If we analyze the binding process in terms of enthalpic and entropic contributions we note that both of them are favourable for the binding at 25°C. However, the entropic term goes from positive (17.2 cal/mol at 25°C) to close to zero (0.4 cal/mol) at 37°C. This results in a shift from a binding process driven preferentially by entropic contributions to an enthalpically promoted one.

**Table 2:** Thermodynamic parameters derived from ITC titrations describing the interaction of (K34)_2_Ni(II) with Tel26, wtTel26 and Tel22 at 37°C in 10 mM Tris, 20 mM KCl, pH 7.5.

	wtTel26	Tel26	Tel22
n (ligands per G4)	1.9±0.1	2.56±0.1	1.6±0.1
Ka*10^−5^ (M^−1^)	4.4±0.7	8.2±2.5	35.1±0.6
ΔH (kcal*mol^−1^)	−7.9±0.2	−5.4±0.2	−4.1±0.1
ΔS (cal*mol^−1^*K^−1^)	0.4	9.6	16.6
-TΔS (kcal*mol^−1^)	−0.1	−3.0	−5.2
ΔG (kcal*mol^−1^)	−8.0±0.1	−8.4±0.2	−9.3±0.1

Reported data are the average of three independent measurements.

Remarkably, this binding profile is generally shared also by Tel26 when titrated with (K34)_2_Ni(II) at 37°C. Indeed, the two binding events described at 25°C are no more clearly dissectible when the titration is performed at higher temperatures (Table 2, Figure S2B). A final stoichiometry close to 2∶1 (the small increase over 2 possibly reflects tendency to ligand aggregation) was preserved but it corresponds to the recognition of two equivalent binding sites. In particular, a comparison of the binding constants at 25°C and 37°C suggests that the lower affinity event recorded at lower temperature is favoured by increasing the working temperature. Indeed, the binding constant for the two equivalent binding events at 37°C, is comparable to the one associated to the high affinity event at 25°C. This can be explained by an incremented accessibility to the second binding site.

**Figure 3:**
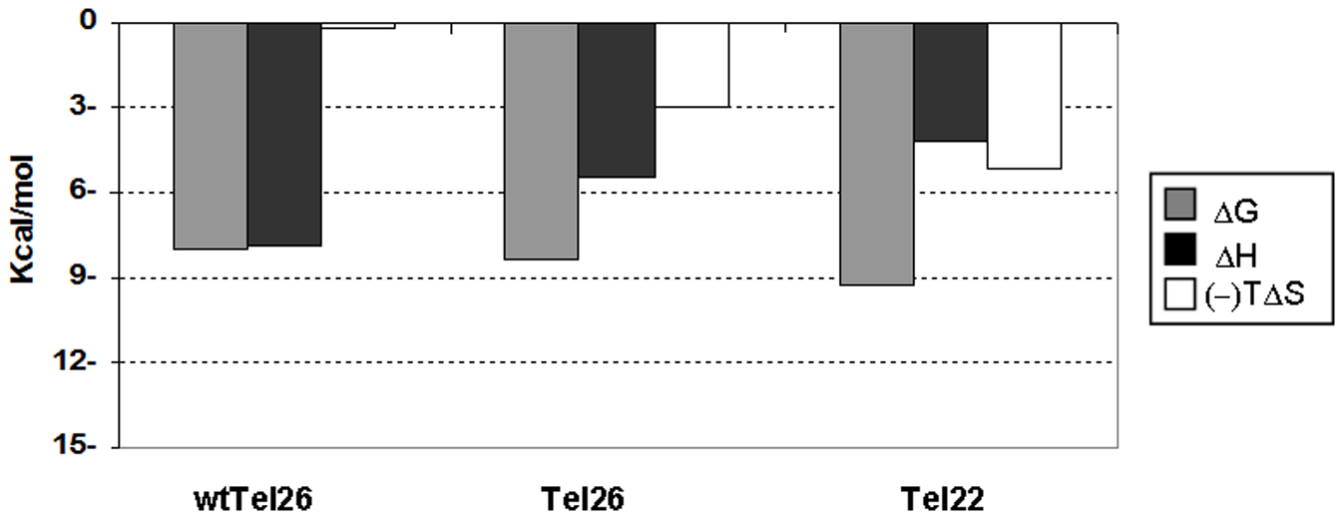
The energetic contribution for (K34)_2_Ni(II) binding towards tested G-quadruplexes is differently modulated. Energetic contributions describing the interaction between tested G-quadruplexes and (K34)_2_Ni(II) at 37°C in 10 mM Tris, 20 mM KCl, pH 7.5. The associated enthalpic variations (ΔH) are shown in black, the entropic ones (-TΔS) in white and the Gibbs energy changes (ΔG) in grey.

As a result, a more effective recognition of (K34)_2_Ni(II) for the Hybrid 1 arrangement was evidenced although with a conserved progressive shift to an entropically less favorable reaction by increasing the working temperature (Figure 3).

At temperatures above 25°C we were able to examine also the Tel22 sequence (Figure S2C). As already seen with Tel26 and wtTel26, the binding process monitored at 37°C was found to be exothermic and could be described by the binding of the metal complex to two equivalent sites. In this case, a stoichiometry lower than 2 might reflect ligand-mediated G-quadruplex-G-quadruplex stacking. Interestingly, Ka value is almost one order of magnitude higher than those observed with the other two tested G-quadruplex templates (Table 2). It is useful to underline that this reflects a relevant entropic term which, associated to the negative ΔH, favourably contributes to the binding process (Figure 3).

To assess the peculiarities of the binding of our metal complex towards G-quadruplex structures, we performed also ITC titrations using the ligand K34 in the absence of the Ni(II) ion (Figure S3). They showed a remarkable suppression of the heat release thus confirming the metal complex as the preferential binder for G-quadruplex structures in agreement with our previously reported data [29].

### Circular Dichroism conformational studies

The calorimetric data clearly indicated that the folding of the analyzed targets highly influences the G-quadruplex binding process of (K34)_2_Ni(II). The capping residues, which are peculiar of the longer tested sequences, might be responsible for such a variation. Indeed, they can represent distinct binding sites. Alternatively, the (K34)_2_Ni(II)-G-quadruplex binding sites can be conserved among the three tested structures and in this case a common structural domain such as the tetrads would represent a reasonable target. As a result, the observed modulation in thermodynamic parameters can at least partly be connected to a different propensity of DNA to undergo structural modifications upon binding. In this connection CD titrations were performed to monitor conformational changes occurring upon ligand-macromolecule binding.

**Figure 4:**
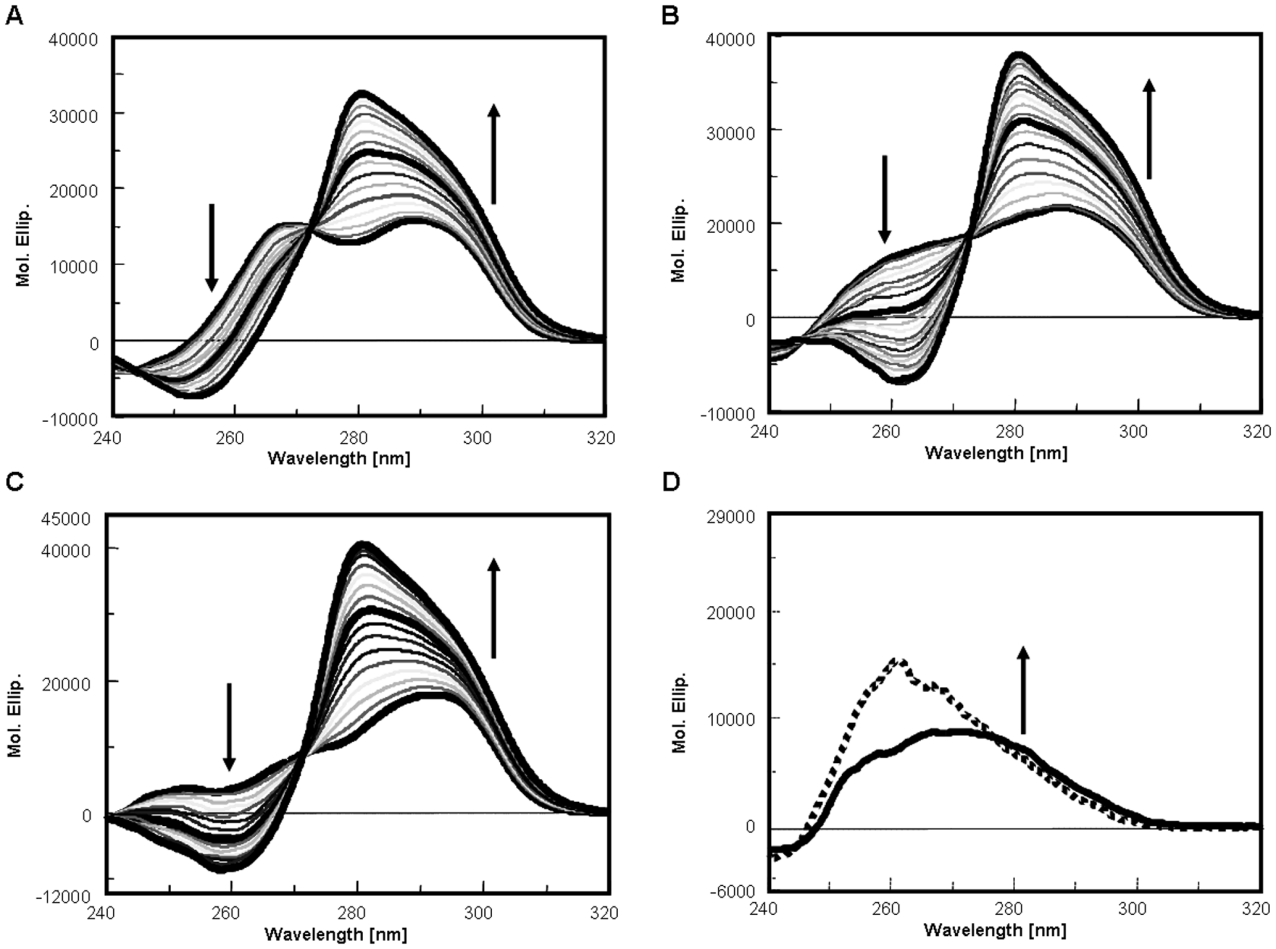
(K34)_2_Ni(II)-G-quadruplex bound form shows conserved CD spectra features. Dichroic spectra of 4 µM Tel26 (Panel A), wtTel26 (Panel B), Tel22 (Panel C) or single strand scrambled scT22 (Panel D) in the presence of increasing concentrations of (K34)_2_Ni(II) at 25°C in 10 mM Tris, 20 mM KCl, at pH 7.5. Arrows indicate changes upon addition of the metal complex, curves in bold indicate spectra of the free oligonucleotide and at 1∶1 and 2∶1 ligand:DNA ratios in panels A–C. In panel D the solid line corresponds to the CD contribution of DNA alone, the dotted line represents the dichroic spectrum at saturating concentrations of (K34)_2_Ni(II).

The recorded dichroic spectra of all tested oligonucleotides are characterized by two positive bands deriving from the 3+1 arrangement, one centered at 290 nm and the other at 265–268 nm. However, they mainly differ in terms of relative intensity and resolution that reflect their distinct folding (Figure 4).

**Figure 5:**
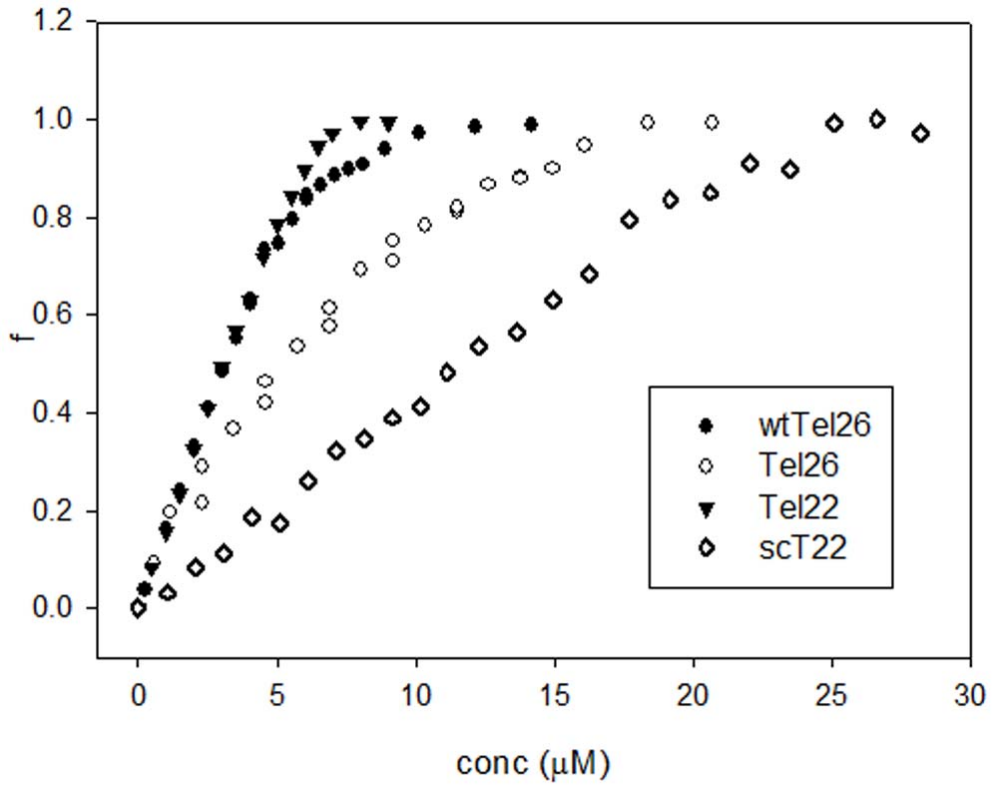
Ligand saturation is modulated by DNA folding. Fraction of rearranged G-quadruplex (f) induced by increasing concentrations of (K34)_2_Ni(II) at 25°C in 10 mM Tris, 20 mM KCl, pH 7.5. Oligonucleotides concentration: 4 µM.

Upon addition of (K34)_2_Ni(II), modifications in the CD spectra of all tested oligonucleotides are detectable even at low ligand versus G-quadruplex ratios (Figures 4 and 5). In agreement with the calorimetric data the signal intensity increased according to one hyperbolic curve and these changes reached saturation at a stoichiometric ratio close to 2∶1.

Due to the spectral properties of tested DNA and of (K34)_2_Ni(II), it is not possible to attribute the observed spectral changes exclusively to the G-quadruplex structural rearrangement. Indeed, overlapping of DNA CD transitions and induced CD of bound metal complex can occur. This prevents a safe quantitative analysis of the binding isotherms derived from the optical signal variations. However, our data support a less efficient process of binding and structural rearrangement for the wtTel26 sequence (Figure 5).

Interestingly, at ligand saturation, CD spectra appear to converge toward a similar signature, characterized by a positive band at 280 nm with a shoulder at 295 nm. This signature is peculiar of the G-quadruplex bound form. Indeed, likely due to its positive charge, (K34)_2_Ni(II) can also interact with different DNA structures. However, the resulting CD spectra of the metal complex bound to non G-quadruplex forming DNAs are quite distinct. As an example, data referring to the interaction of (K34)_2_Ni(II) with an oligonucleotide sequence of the same length and base composition as Tel22 but unable to fold into G-quadruplex is reported in Figure 4D. In this case, a binding constant Ka 2.7 * 10^5^ M^−1^, which involves two bases per metal complex, was observed. A direct comparison of this value with those obtained by ITC in the presence of G-quadruplex folded substrates is not safe. However, as clearly evidenced in Figure 5, the linearly arranged DNA is recognized less efficiently by the tested metal complex.

### Induction and stabilization of G-quadruplex structure

**Figure 6:**
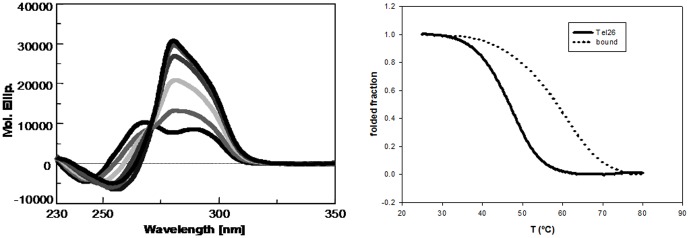
(K34)_2_Ni(II) promotes G-quadruplex folding of the tested telomeric sequences. Dichroic spectra of 4 µM Tel 26 in the presence of increasing concentrations of (K34)_2_Ni(II) at 45 °C (Panel A) and thermal denaturation profiles of the same oligonucleotide (Panel B) recorded at 290 nm in the absence (solid line) or in the presence of (K34)_2_Ni(II) at 2∶1 molar ratio (dotted line) in 10 mM Tris, 20 mM KCl at pH 7.5.

The CD spectral changes associated to the addition of (K34)_2_Ni(II) to G-quadruplex folded telomeric sequences were conserved also when titrations were performed at temperatures above 25°C (37°C and 45°C) (Figure 6). Data referring to the highest temperature are rather interesting. Indeed, the melting profiles obtained while recording the CD signal at 290 nm, indicate that the two longer oligonucleotides, wtTel26 and Tel26, irrespectively of their preferential folding into a unique defined structure, are thermally less stable than Tel22. In particular, their melting temperatures are 44.2°C and 47.2°C, respectively, thus indicating that in solution at 45°C they are partially unfolded. Nevertheless, (K34)_2_Ni(II) was able to produce the same G-quadruplex-bound CD spectra as those recorded at lower temperatures. This suggests that the metal complex is able to shift the DNA structural equilibrium towards a final folded form, which corresponds to the one observed at 25°C. Additionally, this form is stable at physiological temperature. Indeed, melting profiles of (K34)_2_Ni(II):G-quadruplex (molar ratio 2∶1) recorded at 290 nm confirmed a single melting transition shifted to 61.0°C (ΔTm = 13.8°C) using Tel26 and to 59.4°C (ΔTm = 15.2°C) using wtTel26. Distinctly, in the presence of the more thermally stable Tel22 (Tm = 56.2°C), a thermal shift to 66.6°C (ΔTm = 10.4°C) was observed upon metal complex binding. This thermal stabilization is not unexpected since (K34)_2_Ni(II) interacts with G-quadruplex. However, the herein reported increments of DNA melting temperature further sustained the preferential binding of our metal complex to the G-quadruplex folded forms in comparison to the unfolded one.

## Discussion

The thermodynamic signature for the binding of several small molecules to DNA is extensively investigated since it can help understanding the features of the recognition process [39], [40]. However, distinctly from results reported for double stranded DNA, literature data concerning G-quadruplex binders evidenced unpredictable behaviours which do not allow correlating enthalpic/entropic contributions to binding mode [41], [42]. A rational for such variability rests on the polymorphic nature of G-quadruplexes, which is extremely sensitive to buffer composition and DNA sequence. Additionally, different complexes can be formed depending upon ligand nature. As an example, even for a short model sequence like Tel22, although a hybrid-type folding appears to be predominant in potassium containing solutions, the coexistence of different conformations in mutual equilibrium has to be considered [43]. Moreover, different arrangements can be separated by small energy barriers (as in the case of basket-hybrid transition) thus allowing an easy shuffling from one form to the other [44]. To overcome these difficulties, in addition to the reference telomeric sequence Tel22 we used two related sequences, wtTel26 and Tel26, which prevalently fold into two distinct hybrid-type structures. Although only about 75% of the wtTel26 sequence assumes an Hybrid 2 fold whereas essentially Tel26 is fully present in solution in a Hybrid 1 conformation, these two structures do not easily interconvert [32], [33]. Thus, due to their distinct three-dimensional folding they actually represent two structurally different targets for a ligand. In any event it is important to remind that the herein reported thermodynamic analysis represents the sum of multiple contributions, including both binding and structural rearrangement.

The analysis of the recognition process showed that (K34)_2_Ni(II) interacts efficiently with all the tested sequences according to a binding path characterized by a negative ΔH. Additionally, only modest changes in the binding free energy were observed within all the tested templates.

These common hallmarks do not exclude the interaction of (K34)_2_Ni(II) with different portions of the tested G-quadruplex structures. However, the binding stoichiometry provided by ITC and CD analysis indicates two (K34)_2_Ni(II) molecules per G-quadruplex structure. Additionally, dichroic studies showed that, irrespectively of the starting DNA folding, the overall final G-quadruplex-ligand complexes seem to share significant analogies. As above pointed out no DNA structural information can be safely derived by CD spectroscopy. To better dissect the issue of structural similarities among the two complexes, 600 MHz NMR studies were performed (Figure S4). Unfortunately, this powerful technique could be only partly useful due to the large quenching and broadening of the signals due to the presence of the paramagnetic metal center. Nevertheless, comparison of the NMR spectra in the imino protons region clearly suggests that the Tel26 and wtTel26 bound forms share several common peaks (Figure S4B). Interestingly, differences are related to peaks (located at 12.00, 11.05, 10.9 and 11.45, 11,4, 10.55 ppm for H1 and H2, respectively) which result conserved in the free and bound forms. These peaks generally correspond to the imino proton signals relative to the guanines (G11, G17, G6 and G17, G23, G18 for H1 and H2, respectively) located in the central tetrad facing the parallel moiety of the oligonucleotide structure [32], [33].

These results suggest that the binding modes might be conserved among the three tested DNA sequences. In particular, a recognition process which takes place at the terminal G-quartets where π-π stacking interactions can occur between the assembled phenathroline moieties and the exposed aromatic portion of the bases can be inferred.

This shared binding mode can actually help justifying also the differences observed in the binding process. These are:

- a binding pathway which is template dependent. Indeed, at 25 °C, using the Hybrid 1 folded substrate, the binding process occurs through two sequential steps (on two independent sites of the G-quadruplex structure) whereas two equivalent binding sites are identified on the Hybrid 2 structure.
- a higher affinity showed by (K34)_2_Ni(II) for Tel22 in comparison to the longer Tel26 sequences.

It is known from NMR studies that the two tested 26-mer telomeric sequences are selectively stabilized in a defined hybrid folding by the presence of capping structures at the top and bottom of the G-quadruplex core which stacks on the terminal tetrads. Thus it is feasible that these domains impair the accessibility of the metal complex to the target tetrads. Additionally, since these cappings are distinct in Tel26 as compared to wtTel26, it is not odd to assume that the formally conserved binding sites (the G-tetrads) can be seen as similar (as in the case of wtTel26) or different (as in the case of Tel26) by (K34)_2_Ni(II).

Interestingly, in this instance, it appears that a modest increment in the working temperature (thus a modest increment in DNA flexibility) is sufficient to level off such a difference. This points to the need for (K34)_2_Ni(II) to compete with the capping moieties above and below the G-tetrad core to gain access to the planar surface. On the opposite, Tel22, which lacks interfering terminal residues, does not require uncapping to grant exposure of the tetraplex surface, thus exhibiting higher affinity for the metal complex.

By keeping in mind that for Tel26 and wtTel26the capping moieties are actually the structural elements required to select defined topologies among those assumed in solution by Tel22, it turns out reasonable that capping displacement by the ligand can allow the oligonucleotide to rearrange to a conformation best fit for optimal binding.

A final comment is deserved by the entropic contribution to the binding process. Only a limited number of studies deal with the interaction of metal complexes with G-quadruplexes [45]–[47]. In all instances a negative ΔH associated to a predominantly favorable entropic contribution was reported. Due to the charged nature of metal complexes, this likely reflects significant DNA counterions release produced upon binding in addition to solvation and hydrogen bonding effects. However, with our tested sequences, upon increasing the working temperature, an enthalpy–entropy compensation occurred (more negative ΔH, less positive ΔS), a well documented behaviour typical of ligand-receptor binding [48]. Interestingly, this effect is more pronounced when working close to the G-quadruplex melting temperature (Figure S5 and Table S1). Since our metal complex is actually able to induce refolding of the melted oligonucleotides, the compensation effect may be reinforced by the introduction of structural constraints in the nucleic acid upon interaction with (K34)_2_Ni(II).

In conclusion, our study demonstrated how different G-quadruplex structures, produced by recurrence of a G-quadruplex forming sequence in closely related oligonucleotides, do not grant unique quantitative information on ligand binding. Consequently, an anticipation of possible pharmacological implications of the recognition process is very hard to adequately rationalize, even not considering metabolic effects.

Here, we show how the binding process can be thermodynamically affected not only in terms of enthalpy and entropy changes but also in terms of binding site equivalence. We have seen that the uncapped Tel22 can bind to a ligand with higher affinity than the structurally constrained 26-mer sequences, that increasing temperature leads to similar binding strength notwithstanding the exothermic nature of the process, that individual binding sites can be turned from similar to dissimilar on the same template for the same ligand just by slightly adjusting temperature conditions. Combination of these events allows distinct G-quadruplex template structures to adopt similar arrangements when in complex with an effective small molecule binder.

The more we learn, the less simple is foreseeing thermodynamics and stereochemistry of induced fit effects and the consequent biological/pharmacological implications of an apparently straightforward process such as ligand G-quadruplex recognition.

## Supporting Information

Figure S1 **UV spectra of 300 µM (grey solid line) and 5 µM (black dotted line) of (K34)_2_Ni(II) in 10 mM Tris, 20 mM KCl, pH 7.5.**
(DOC)

Figure S2 **ITC profiles corresponding to the titration of 25 µM wtTel26 (Panel A), Tel26 (Panel B) or Tel22 (Panel C) with (K34)2Ni(II) at 37°C in 10 mM Tris, 20 mM KCl, pH 7.5.** Raw ITC data (top panel) and binding isotherms (bottom panel).
(DOC)

Figure S3 **ITC profiles corresponding to the titration of 25 µM Tel26 with (K34)2Ni(II) (Panel A) or K34 (Panel B) at 37°C in 10 mM TRIS, 20 mM KCl, at pH 7.5.** Raw ITC data (top panel) and binding isotherms (bottom panel).
(DOC)

Figure S4 **Imino proton region of the 1D 1H NMR spectrum of Tel26 (grey) and wtTel26 (black) in 20 mM K+ solution, pH 7.4, recorded before (PANEL A) after addition of 2 equivalents of (K34)2NI(II) (PANEL B) at 25°C.**
(DOC)

Figure S5 **ITC profiles corresponding to the titration of 18 µM Tel22 with (K34)2Ni(II) at 45 °C in 10 mM TRIS, 20 mM KCl, at pH 7.5.** Raw ITC data (top panel) and binding isotherms (bottom panel).
(DOC)

Table S1 **Thermodynamic parameters derived from ITC titrations describing the interaction of (K34)2Ni(II) with Tel22 at 45°C in 10 mM Tris, 20 mM KCl, pH 7.5.**
(DOC)
