# Supplementary material for: Effect of G-Quadruplex Polymorphism on the Recognition of Telomeric DNA by a Metal Complex
Source: PLoS One. 2013 Mar 13;8(3):e58529. doi: 10.1371/journal.pone.0058529 (PMC3596309; doi:10.1371/journal.pone.0058529)
Supplement: Figure S1 — UV spectra of 300 µM (grey solid line) and 5 µM (black dotted line) of (K34)2Ni(II) in 10 mM Tris, 20 mM KCl, pH 7.5. (DOC) [file pone.0058529.s001.doc]

Figure S1. UV spectra of 300 µM (grey solid line) and 5 µM (black dotted line) of (K34)_2_Ni(II) in 10 mM Tris, 20 mM KCl, pH 7.5.
