# Supplementary material for: Effect of G-Quadruplex Polymorphism on the Recognition of Telomeric DNA by a Metal Complex
Source: PLoS One. 2013 Mar 13;8(3):e58529. doi: 10.1371/journal.pone.0058529 (PMC3596309; doi:10.1371/journal.pone.0058529)
Supplement: Figure S2 — ITC profiles corresponding to the titration of 25 µM wtTel26 (Panel A), Tel26 (Panel B) or Tel22 (Panel C) with (K34)2Ni(II) at 37°C in 10 mM Tris, 20 mM KCl, pH 7.5. Raw ITC data (top panel) and binding isotherms (bottom panel). (DOC) [file pone.0058529.s002.doc]

Figure S2. ITC profiles corresponding to the titration of 25 µM wtTel26 (Panel A), Tel26 (Panel B) or Tel22 (Panel C) with (K34)_2_Ni(II) at 37 ºC in 10 mM Tris, 20 mM KCl, pH 7.5. Raw ITC data (top panel) and binding isotherms (bottom panel).
