# Supplementary material for: Effect of G-Quadruplex Polymorphism on the Recognition of Telomeric DNA by a Metal Complex
Source: PLoS One. 2013 Mar 13;8(3):e58529. doi: 10.1371/journal.pone.0058529 (PMC3596309; doi:10.1371/journal.pone.0058529)
Supplement: Figure S4 — Imino proton region of the 1D 1H NMR spectrum of Tel26 (grey) and wtTel26 (black) in 20 mM K+ solution, pH 7.4, recorded before (PANEL A) after addition of 2 equivalents of (K34)2NI(II) (PANEL B) at 25°C. (DOC) [file pone.0058529.s004.doc]

A


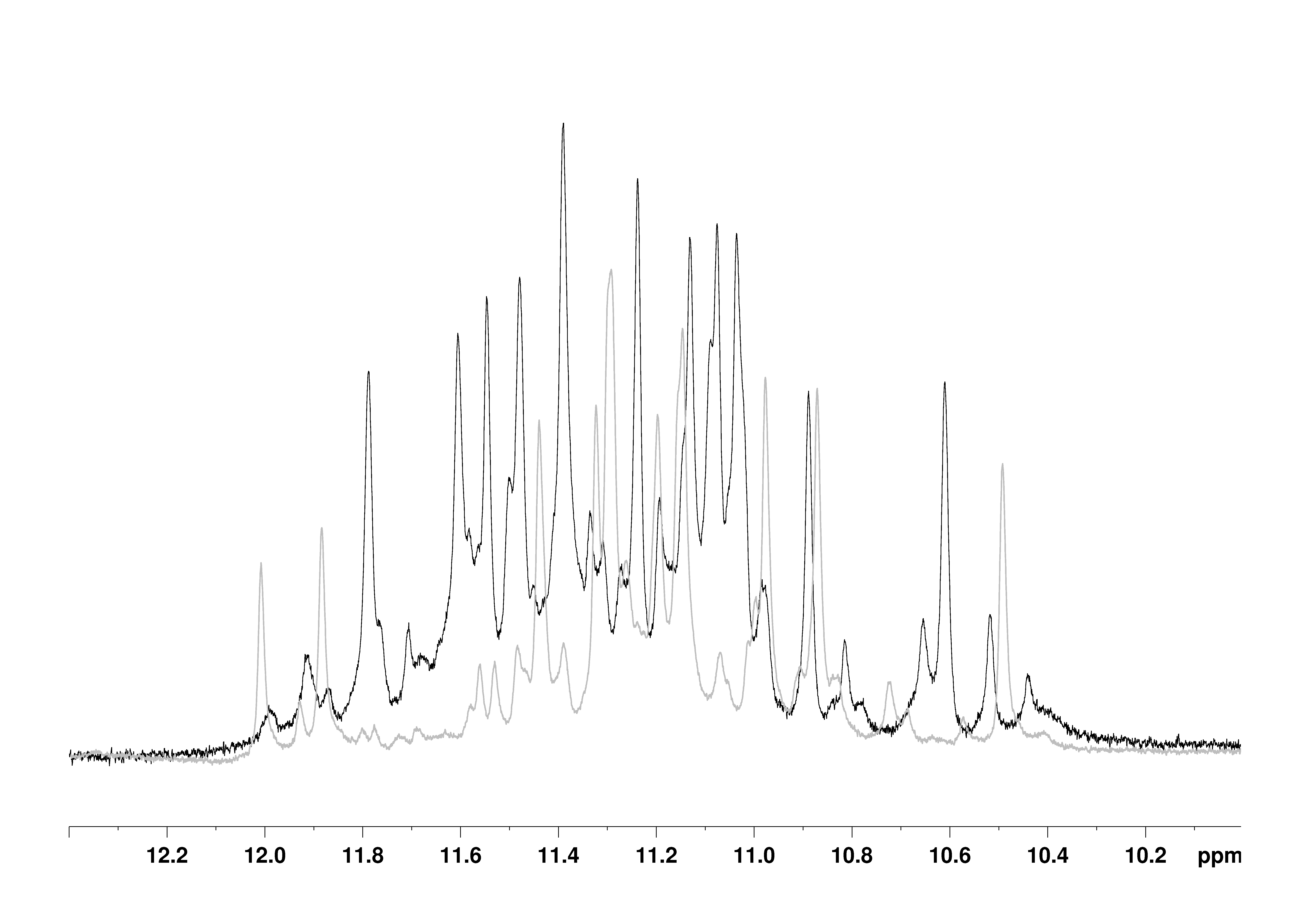


B


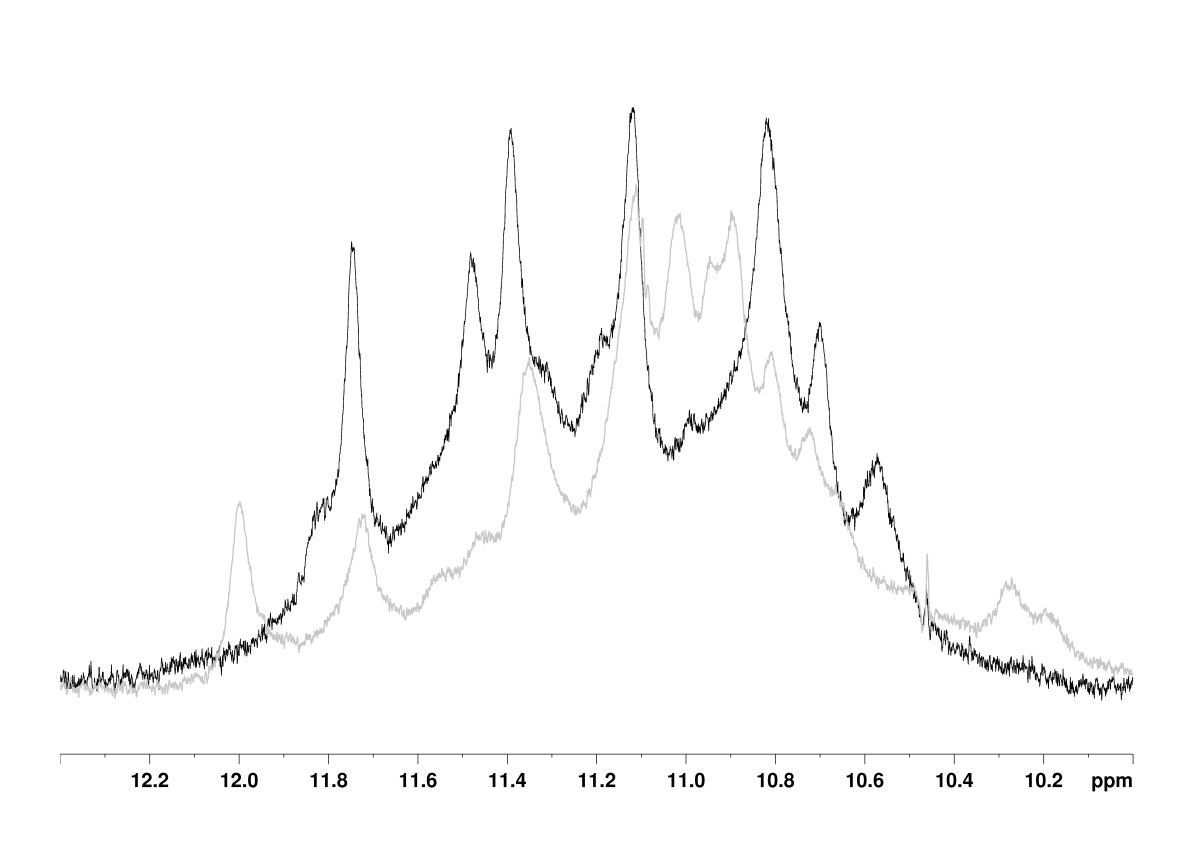


Figure S4. Imino proton region of the 1D ^1^H NMR spectrum of Tel26 (grey) and wtTel26 (black) in 20 mM K^+^ solution, pH 7.4, recorded before (PANEL A) after addition of 2 equivalents of (K34)_2_NI(II) (PANEL B) at 25 °C.
