# Supplementary material for: Effect of G-Quadruplex Polymorphism on the Recognition of Telomeric DNA by a Metal Complex
Source: PLoS One. 2013 Mar 13;8(3):e58529. doi: 10.1371/journal.pone.0058529 (PMC3596309; doi:10.1371/journal.pone.0058529)
Supplement: Figure S5 — ITC profiles corresponding to the titration of 18 µM Tel22 with (K34)2Ni(II) at 45 °C in 10 mM TRIS, 20 mM KCl, at pH 7.5. Raw ITC data (top panel) and binding isotherms (bottom panel). (DOC) [file pone.0058529.s005.doc]

Figure S5. ITC profiles corresponding to the titration of 18 µM Tel22 with (K34)_2_Ni(II) at 45 ºC in 10 mM TRIS, 20 mM KCl, at pH 7.5. Raw ITC data (top panel) and binding isotherms (bottom panel).
