# Supplementary material for: Effect of G-Quadruplex Polymorphism on the Recognition of Telomeric DNA by a Metal Complex
Source: PLoS One. 2013 Mar 13;8(3):e58529. doi: 10.1371/journal.pone.0058529 (PMC3596309; doi:10.1371/journal.pone.0058529)
Supplement: Table S1 — Thermodynamic parameters derived from ITC titrations describing the interaction of (K34)2Ni(II) with Tel22 at 45°C in 10 mM Tris, 20 mM KCl, pH 7.5. (DOC) [file pone.0058529.s006.doc]

Table S1. Thermodynamic parameters derived from ITC titrations describing the interaction of (K34)_2_Ni(II) with Tel22 at 45 °C in 10 mM Tris, 20 mM KCl, pH 7.5.

| ***parameter*** | ***value*** |
| --- | --- |
| **n (ligands per G4)** | 1.9 ± 0.1 |
| **Ka*10^-5^ (M^-1^)** | 11.0 ± 2.0 |
| Δ**H (kcal*mol^-1^)** | -5.6 ± 0.2 |
| Δ**S (cal*mol^-1^*K^-1^)** | 10.9 |
| **-T**Δ**S (kcal*mol^-1^)** | -3.5 |
| Δ**G (kcal*mol^-1^)** | -8.8 ± 0.1 |
